# Supplementary material for: Inferring single-cell resolution spatial gene expression via fusing spot-based spatial transcriptomics, location, and histology using GCN
Source: Brief Bioinform. 2024 Dec 2;26(1):bbae630. doi: 10.1093/bib/bbae630 (PMC11645551; doi:10.1093/bib/bbae630)
Supplement: Supplementary_Materials_Final_bbae630 [file supplementary_materials_final_bbae630.pdf]

Supplementary Materials for

Inferring single-cell resolution spatial gene expression via fusing spot-based spatial transcriptomics, location and histology using GCN

Supplementary Notes

1. Baseline methods

We selected several state-of-the-art methods in super-resolution task that are representative of the following:

- **iStar** [1] models super-resolution gene expression from hierarchical histological features using a feedforward neural network. This method is divided into two parts: the HIPT model [2] is used to extract hierarchical histological features, and then a feedforward neural network is used to predict super-resolution gene expression. iStar emphasizes multi-layer histological feature extraction. Each superpixel contains not only local cell feature information, but also global relationship information in the entire histology image.
- **XFuse** [3] integrates Spatial transcriptomics (ST) data and histology images using a deep generative model to infer super-resolution gene expression profiles. This method considers spatial gene expression and histological image data as observable effects of potential tissue states, and maps image data to potential states through a recognition neural network. XFuse performs well in the top-ranked highly expressed genes, but is misled by intense morphological similarities between different regions in histology image, resulting in poor prediction of low-expression regions of genes.
- **TESLA** [4] generates high-resolution gene expression profiles based on Euclidean distance metric, which considers the similarity in physical locations and histology image features between superpixels and measured spots. TESLA generates super-resolution gene expression based on the assumption that the expression patterns for spatially variable genes are correlated with histology image features. Therefore, it is likely to perform poorly on non-spatially variable genes.
- **STAGE** [5] to generate gene expression data for unmeasured spots or points from Spatial Transcriptomics with a spatial location-supervised Auto-encoder GEnerator by integrating spatial information and gene expression data. STAGE was originally designed to predict gene expression at spot gaps, but we were also able to obtain super-resolution gene expression using STAGE by converting spatial coordinates from the spot level to the superpixel level.

There are other excellent computational methods for enhancing the resolution of ST data, such as BayesSpace [6] and HisToGene [7]. But we did not compare scstGCN with them because BayesSpace separates a spot into several sub-spots and cannot impute gene expression for unmeasured locations; HisToGene merely interpolated the gaps between spots and did not enhance the gene expression levels to single-cell resolution.

2. Implementation Details

For all baselines, default parameters from the original papers were used, and all experiments were executed on a single NVIDIA RTX 3090 GPU using Python 3.11.5 and Pytorch (version 2.1.1). The training protocol was established for 500 epochs and a learning rate set at 0.0001. The batch size is set to the minimum value between 128 and the integer division of number of spot by 16. In the multimodal feature mapping extractor of scstGCN, the ViT architecture utilizes a self-pretrained model, UNI [8], which uses DINOv2 to pretrain on over 100 million images from diagnostic H&E-stained WSIs spanning 20 major tissue types. In our GCN module, the number of nodes in each subgraph is determined based on specific features of the dataset. For example, Visium and ST data have spot sizes of 55 μm and 200 μm, respectively, while the size of a single cell is approximately 8 μm. Therefore, the number of nodes in the subgraph is set to 7×7 and 29×29, which exactly covers the entire spot.

3. Evaluation metrics

To evaluate the accuracy of predicted single-cell resolution gene expression profiles by scstGCN and other baseline methods, for each gene, we treated both the ground truth and predicted profiles as images, where the image intensity was normalized to the range of $[0.0, 1.0]$, and then the prediction accuracy was measured by all evaluation metrics.

In this study, we employ the Root Mean Square Error (RMSE) and Structural Similarity Index Measure (SSIM) to compare the predicted super-resolution gene expression with the observed ground truth from Xenium datasets. To calculate the RMSE, the ground truth and the predicted gene expression profiles were flattened into vectors, with the RMSE was equal to the Euclidean distance between the two vectors. The calculation formula of RMSE is as follows:

$$\textbf{RMSE} = \sqrt{\frac{1}{n}\sum_{i=1}^{n}(u_i - v_i)^2}, \tag{1}$$

where u_i and v_i denote the flattened super-resolution gene expression obtained by prediction and the flattened ground truth, respectively. n denotes the length of flattened vectors. RMSE is a direct and quick metric for assessing the accuracy of predictions for any vectorizable outcome. However, for image data, RMSE ignores spatial context within the image. Therefore, we also calculate SSIM, a measure of image similarity widely used in tasks such as super-resolution and medical imaging. A higher SSIM indicates

greater similarity between two images. The SSIM can be expressed as follows:

$$\mathbf{SSIM} = \frac{\left(2\mu_x\mu_y + C_1\right)\left(2\mathbf{conv}(x,y) +_2\right)}{\left(\mu_x^2 + \mu_y^2 + C_1\right)\left(\sigma_x^2 + \sigma_y^2 + C_2\right)}, \tag{2}$$

where x and y represent the ground truth and the predicted super-resolution gene expression, respectively. u_x, u_y denote the mean of x, y. σ_x, σ_y denote the standard deviation of x, y. **conv** denotes covariance. C_1 and C_2 are set to 0.01 and 0.03, respectively.

When evaluating metrics on Visium or from other platforms data without super-resolution gene expression as a label, after a post-processing step, we used the RMSE, Pearson correlation coefficient (PCC), and Mean Absolute Error (MAE) to evaluate the performance of super-resolution gene expression predictions. The PCC has a range of values $[-1, 1]$. It measures the strength of the linear relationship between two variables by dividing the covariance of the two variables by the product of their respective standard deviations:

$$\mathbf{PCC} = \frac{\mathbf{conv}(x,y)}{\sigma(x)\sigma(y)}. \tag{3}$$

MAE represents the simple average of the absolute errors between predicted and observed values, exhibits lower sensitivity to outliers, and equally considers the magnitude of all errors. Similar to RMSE, the observed values and the predicted values were flattened into vectors before calculating the MAE.The MAE can be expressed as follows:

$$\mathbf{MAE} = \frac{1}{n}\sum_{i=1}^{n}\left|u_i - v_i\right|. \tag{4}$$

In super-resolution tasks, PCC is not a common metric because it is sensitive to abnormal noise anomalies. PCC is a common evaluation criterion in spot-based ST data, as the number of spots in low resolution spot-based ST data typically ranges from hundreds to thousands. In super-resolution tasks, the number of superpixels can reach hundreds of thousands or even millions, PCC is not suitable in high noise magnitude in the super-resolution gene expression. Therefore, in the experimental evaluation of post-based ST data such as DLPFC, we used PCC as the Evaluation metric, whereas in Xenium data, we did not use PCC to measure performance.

Supplementary Figures

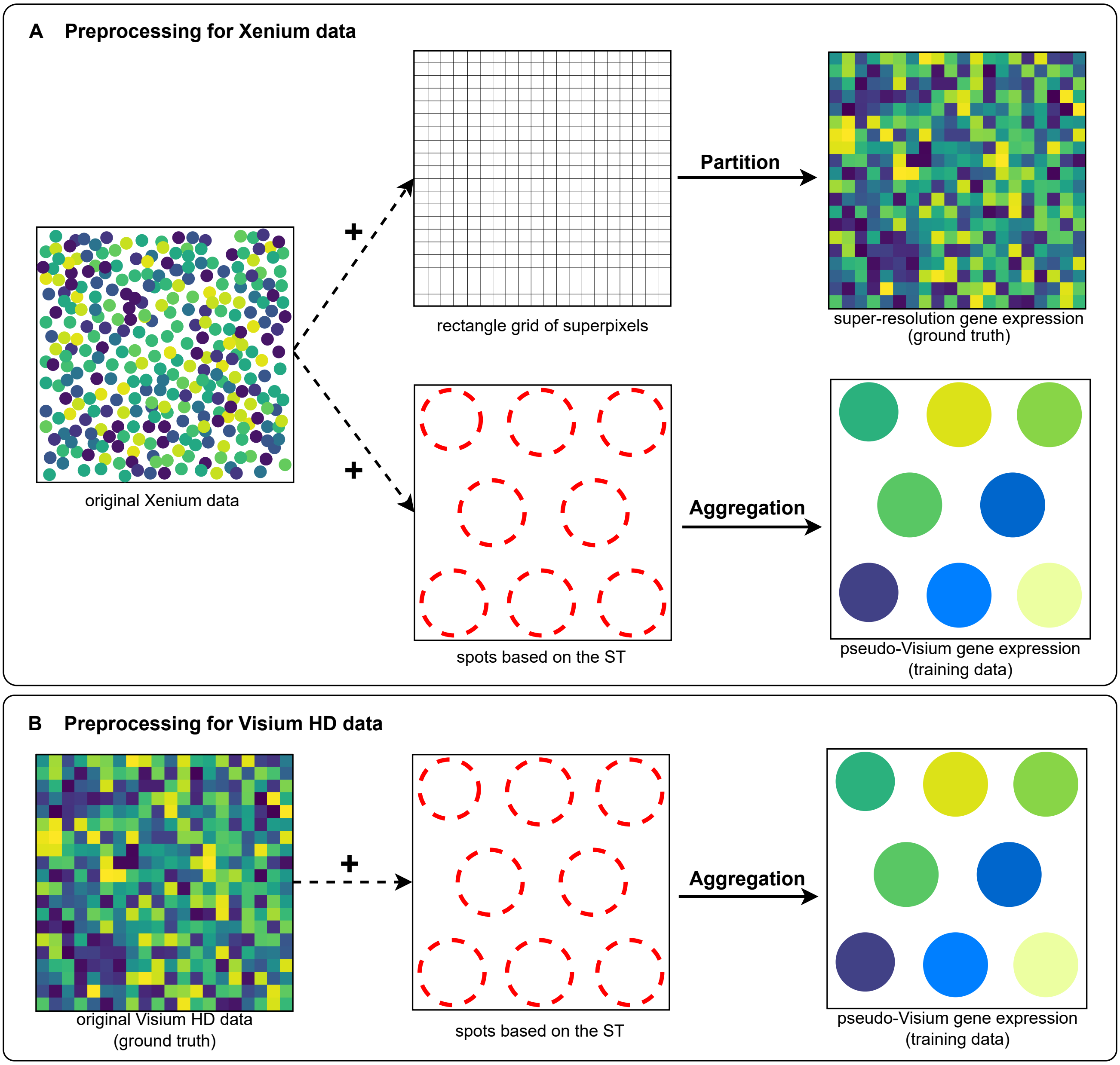

Figure S1. The steps for generating and processing the datasets in Xenium and Visium HD datasets. **(A)** Preprocessing for Xenium data. **(B)** Preprocessing for Visium HD data.And the "post-processing" step in DLPFC data is basically the same as this process.

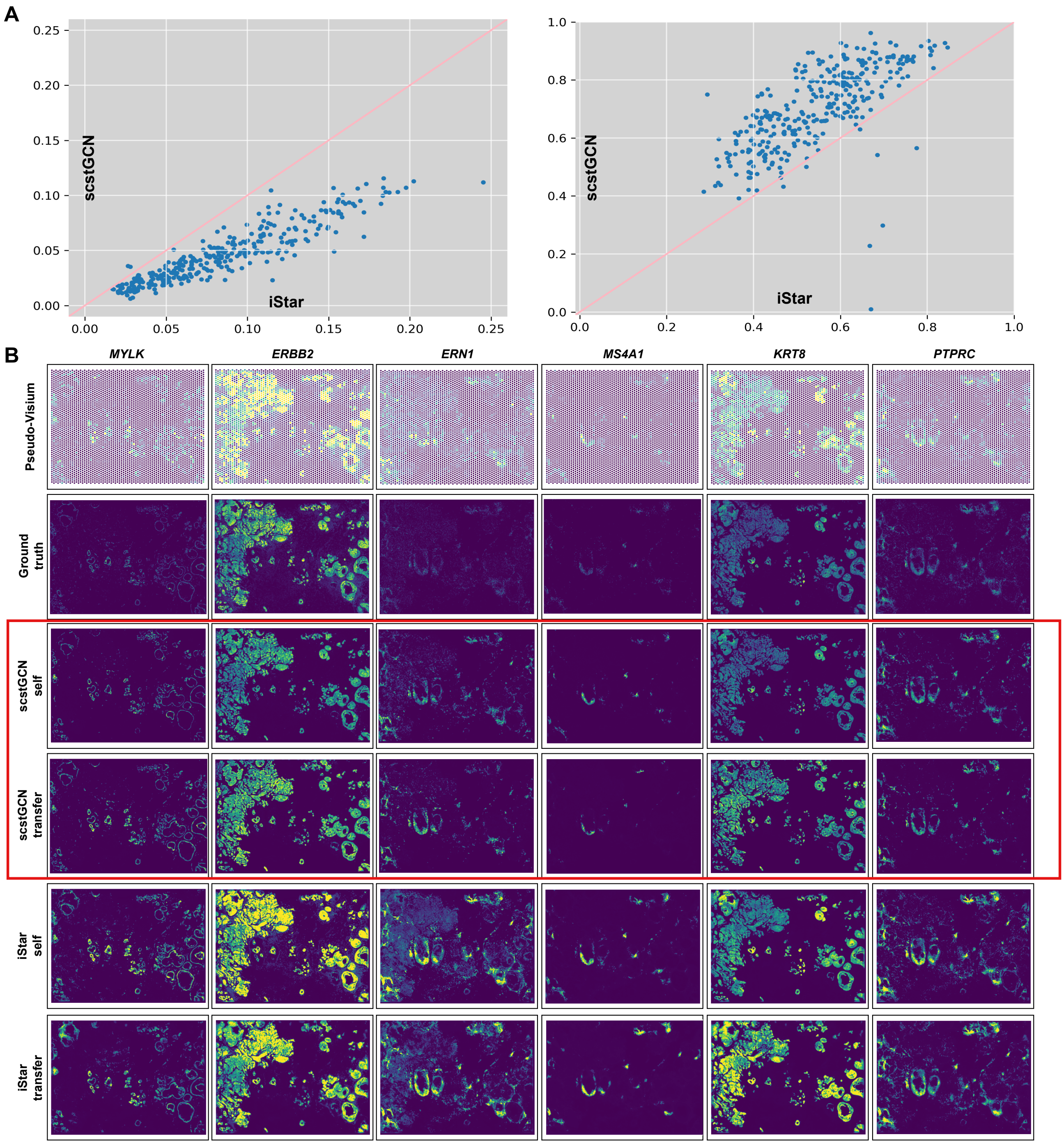

Figure S2. Numerical evaluation and spatial gene visualization results of scstGCN and state-of-the-art method iStar in transfer learning. For transfer learning, the pseudo-Visium data of HBC_S1R2 from human breast cancer (HBC) Xenium data were used as the training data, and super-resolution gene expression profiles was obtained on HBC_S1R1 using only its histological image as the input. **(A)** Scatter plots of RMSE (left) and SSIM (right) between the ground truth and the super-resolution gene expression predicted by the scstGCN and iStar for the all 313 genes. Each dot represents one of the 313 genes. **(B)** Spatial expression analysis of multiple groups of genes with different spatial patterns in HBC_S1R1 data. The analysis are based on pseudo-Visium data, super-resolution ground truth, and predicted data using scstGCN and iStar by weakly-supervised learning and transfer learning, respectively. Each column corresponds to a gene, with the first two rows from the top displays the pseudo-Visium and ground truth, while the subsequent rows show the predicted data using different methods.

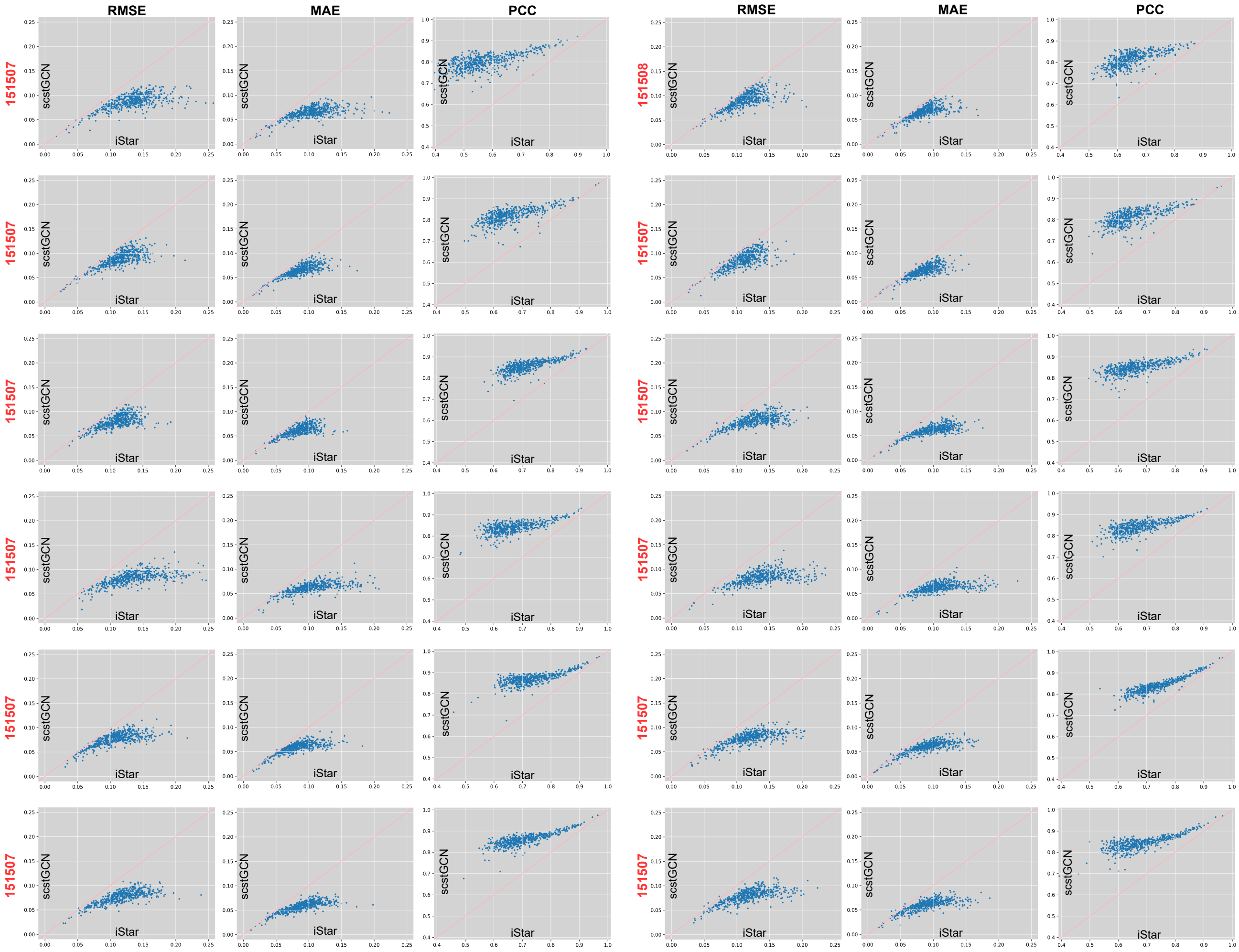

Figure S3. Numerical evaluation of prediction accuracy between the original spot-level gene expression and "spot-level" gene expression obtained from the enhanced expression generated by scstGCN and state-of-the-art method iStar. The prediction accuracy of scstGCN and iStar was measured using root Mean Square Error (RMSE), Mean Absolute Error (MAE) and Pearson correlation coefficient (PCC) measurements for 1000 highly variable genes in each of all 12 sections from the human dorsolateral prefrontal cortex (DLPFC) tissue data. In each scatter plot, a dot represents a gene. The results show that scstGCN achieved better performance in all sections.

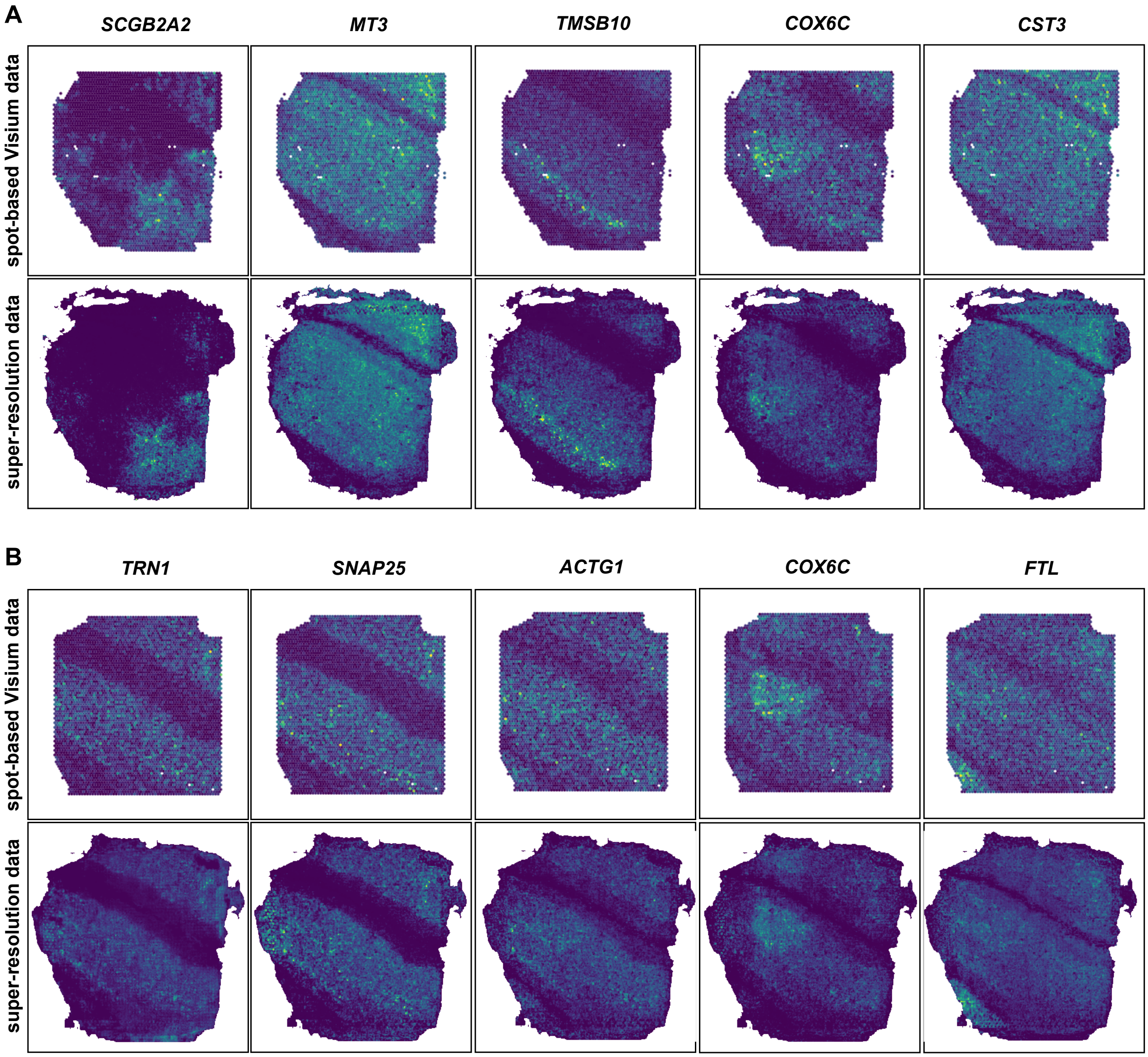

Figure S4. scstGCN can not only improve the resolution within the measured spot, but also predict single-cell resolution gene expression in non measured spot areas of histological images. As long as the model is trained at all measured spots, the super-resolution gene expression of all tissue regions can be predicted based solely on histological imag. **(A)** and **(B)** represent the spatial visualization of several genes having different spatial patterns for the spot-based Visium data and predicted super-resolution data by scstGCN in section 151507 and 151510, respectively.

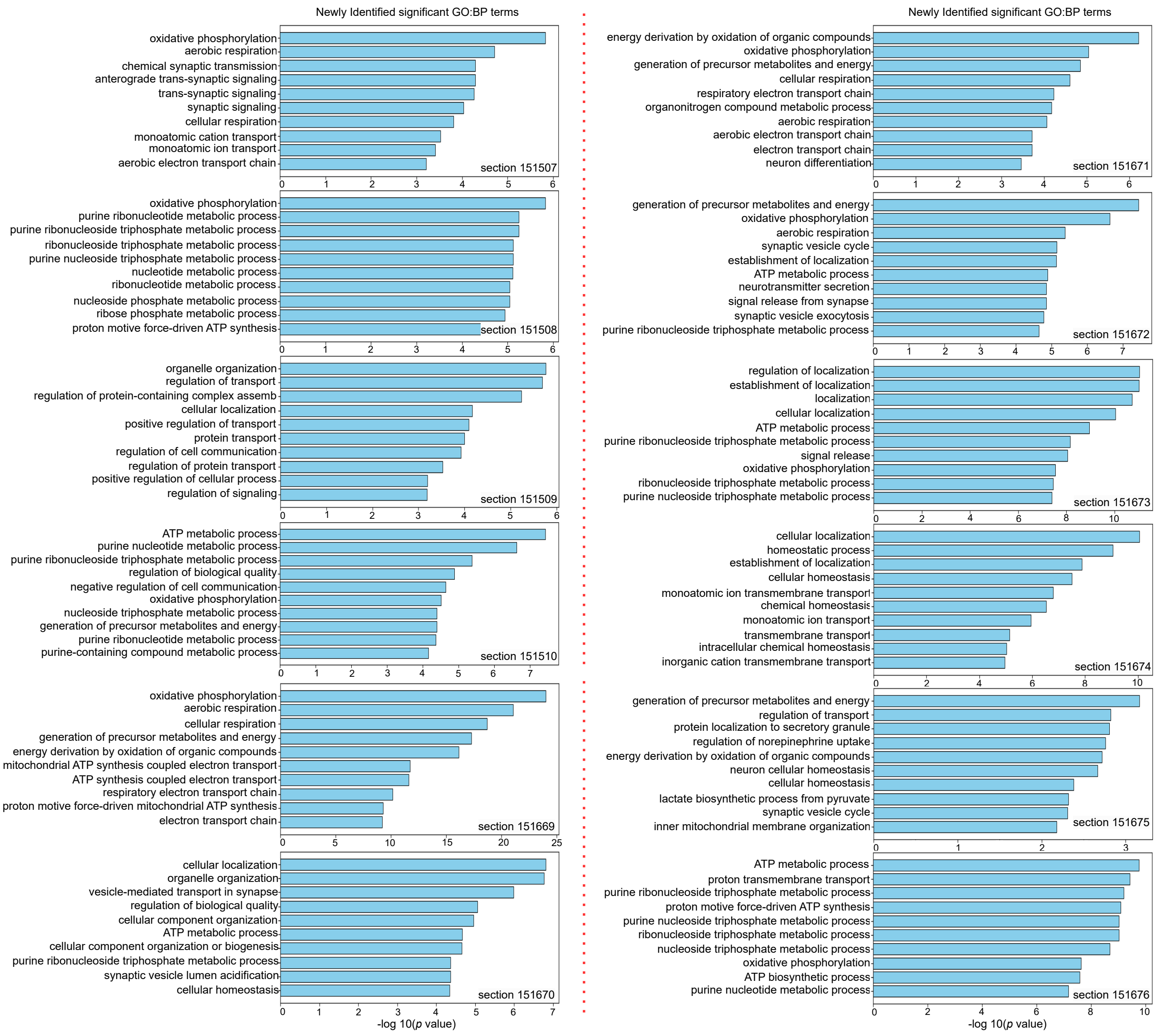

Figure S5. Top-10 significant GO:BP terms were only identified in the super-resolution data predicted by scstGCN in all sections from DLPFC tissue.

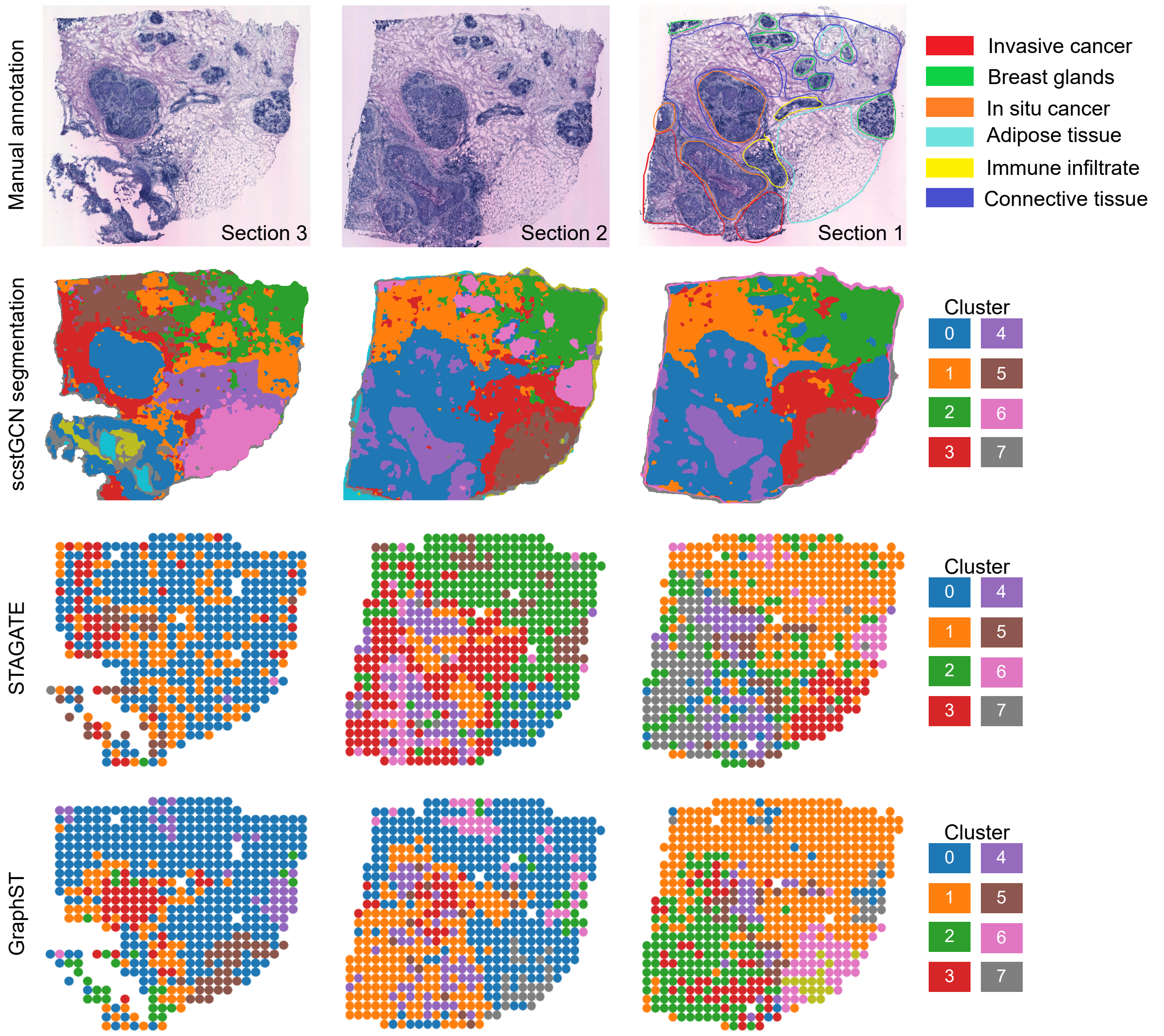

Figure S6. scstGCN adeptly performs super-resolution segmentation of tissue structures with fine granularity. STAGATE and GraphST only perform spatial domain identification on the raw data at spot-level with coarse granularity.

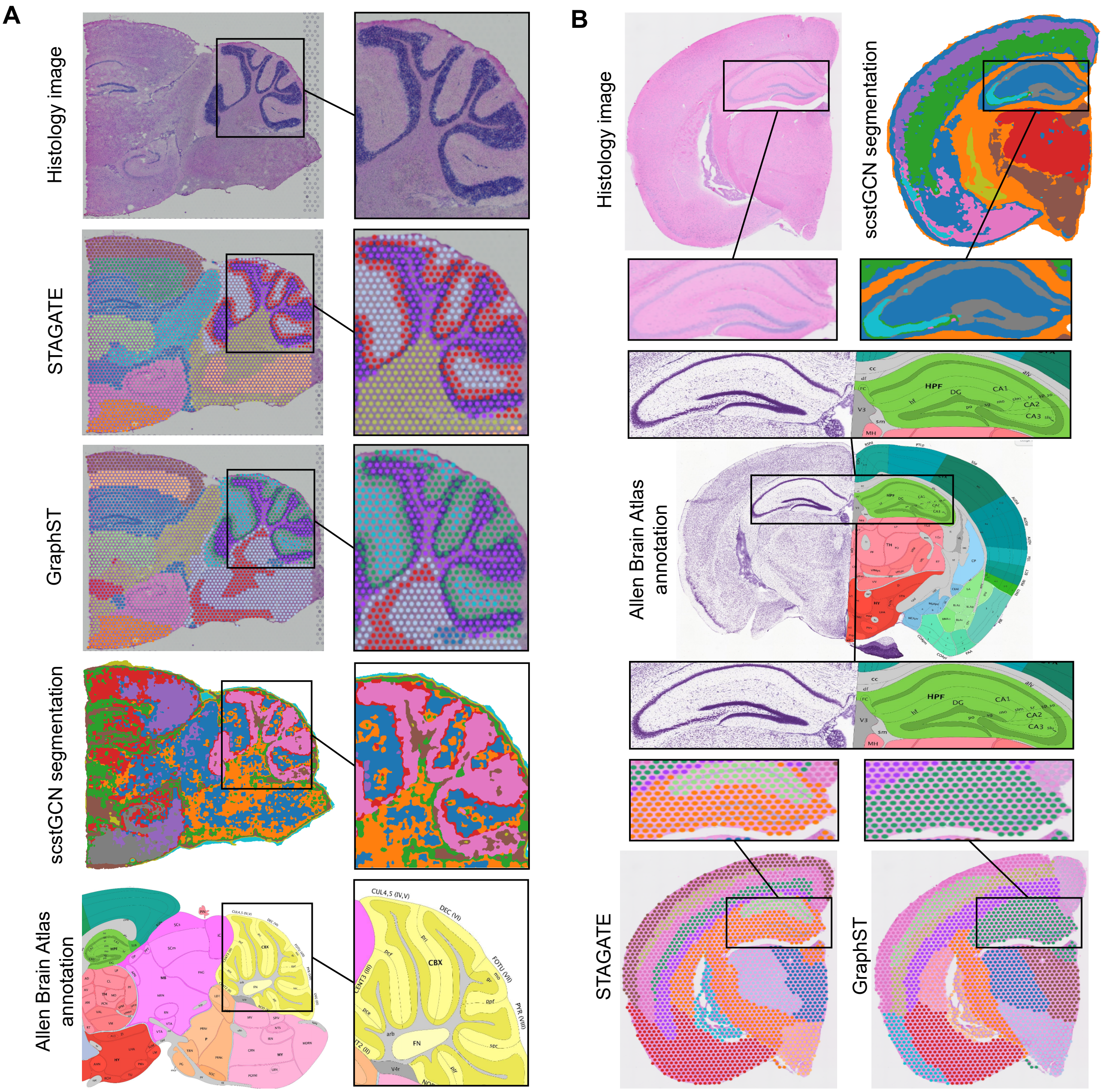

Figure S7. Comparison of tissue segmentation on MBC and MBS data using spatial clustering methods STAGATE and GraphST,and scstGCN method capable of tissue segmentation at super-resolution respectively. **(A)** mouse brain coronal cut data. **(B)** mouse brain sagittal cut posterior data.

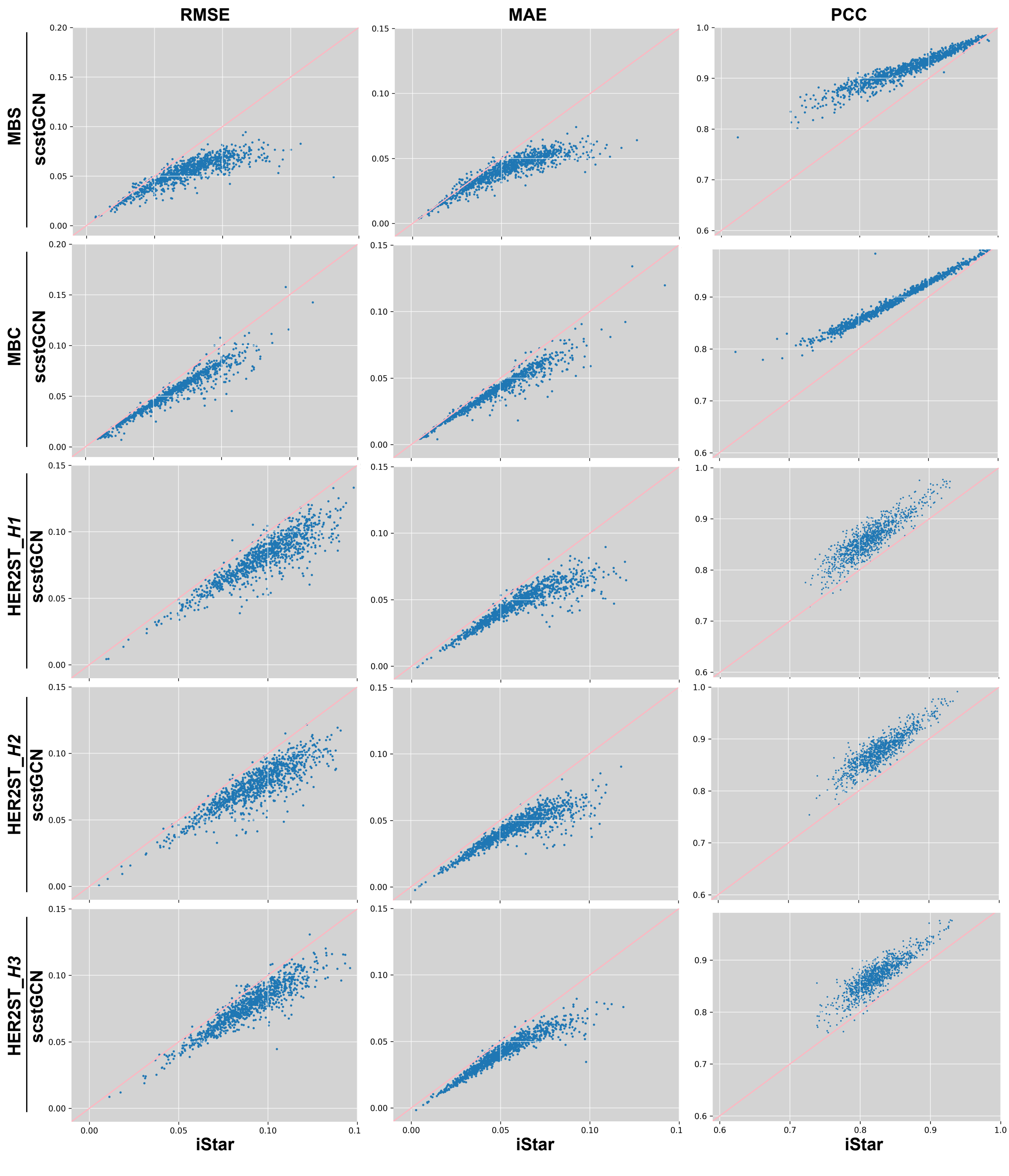

Figure S8. Numerical evaluation of predictive performance of scstGCN and state-of-the-art method iStar on the human HER2 positive breast cancer (HER2ST), mouse brain sagittal cut (MBS) and mouse brain coronal cut (MBC) datasets. For HER2ST data, we considered three consecutively cut sections from sample H. Each row corresponds to a data, and each column corresponds to one of the evaluation metrics of RMSE, MAE, and PCC. In each scatter plot, a dot represents one of the 1000 highly variable genes. The results show that scstGCN outperforms the state-of-the-art method iStar across all datasets.

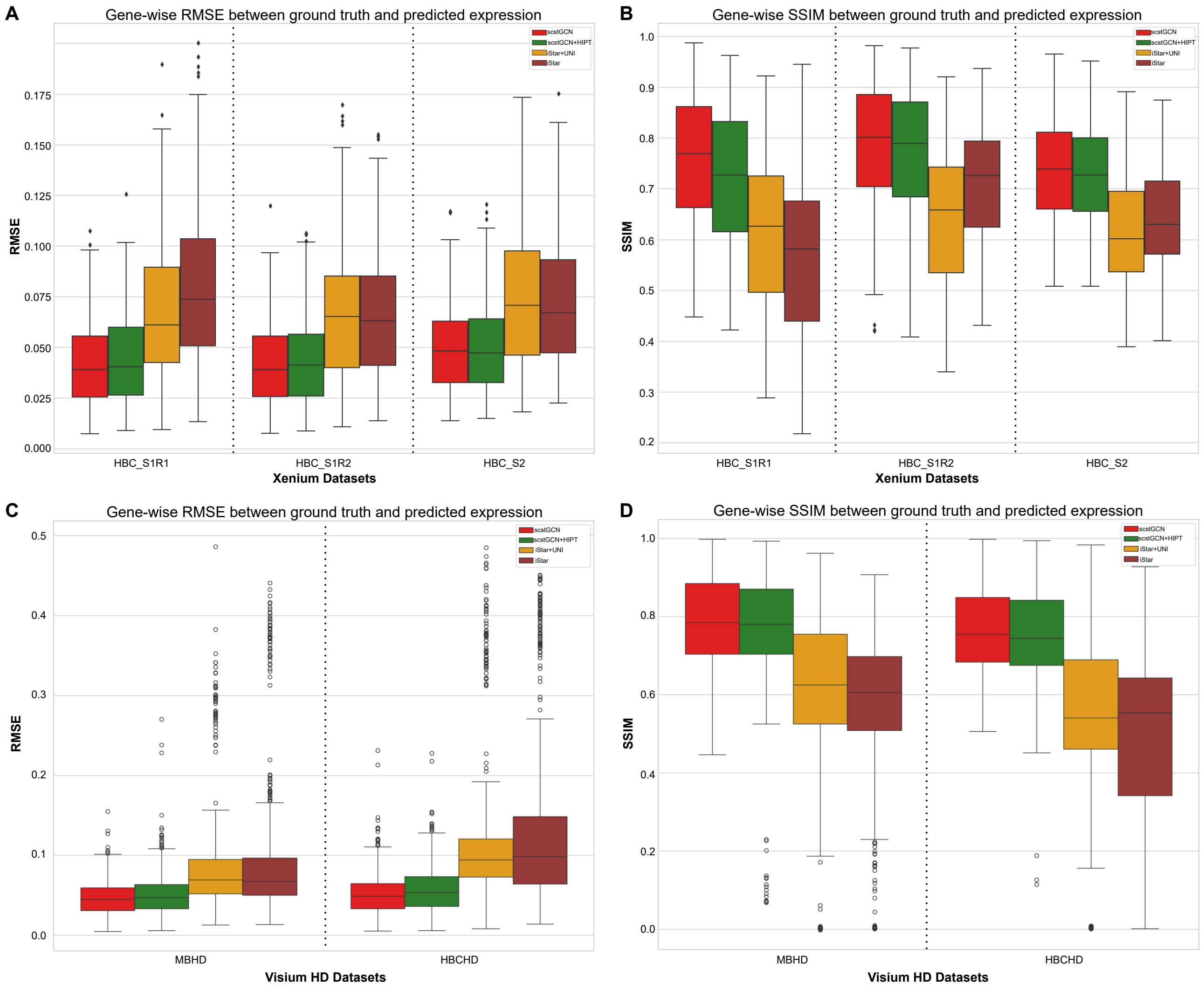

Figure S9. The performance difference between scstGCN and iStar on HBC data from Xenium platform and MBHD, HBCHD data from Visium HD platform, under the condition that both use the same underlying Vision Transformer(UNI or HIPT). **(A, B)** Calculation result in RMSE and SSIM of HBC datasets. **(C, D)** Calculation result in RMSE and SSIM of MBHD and HBCHD datasets.

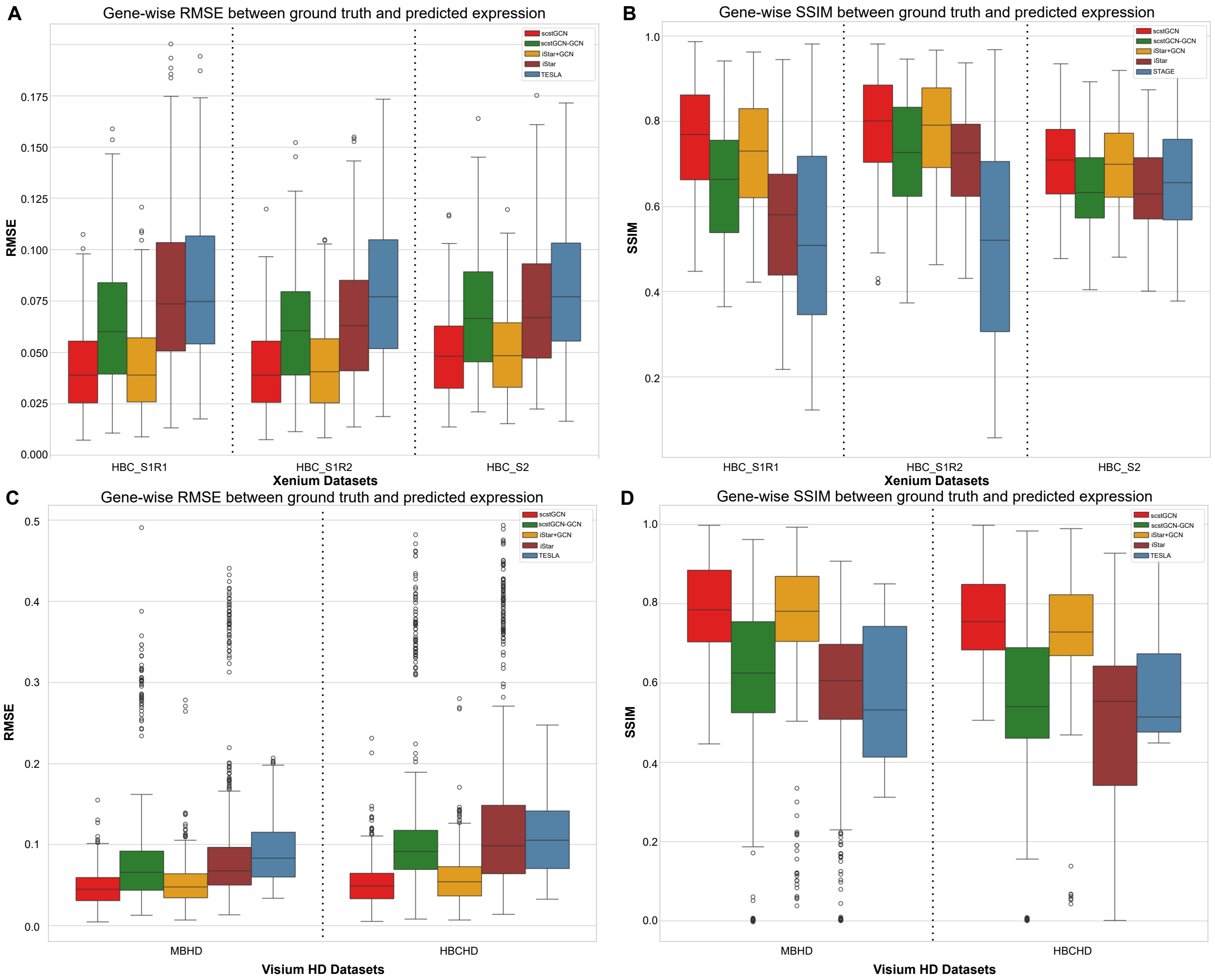

Figure S10. Comprehensive performance evaluation of the GCN module on Xenium and Visium HD datasets. "red" and "orange" boxes represent the introduction of GCN module to establish complex communication relationships between neighboring cells. **(A, B)** Calculation result in RMSE and SSIM of HBC datasets. **(C, D)** Calculation result in RMSE and SSIM of MBHD and HBCHD datasets. The improvement of the single-cell resolution gene expression prediction accuracy of scstGCN compared with iStar is greater than that of iStar compared with TESLA, which serves as the best benchmark against iStar.

Supplementary Tables

Table S1. Summary of ST datasets used in this study.

Datasets	Species	Tissue	Size	Sections	Spots/Cells	Genes	Platform	Data source
HBC	Human	Breast cancer	single-cell	3	167,780 cells (Section 1) 118,752 cells (Section 2) 142,272 cells (Section 3)	313 genes (Section 1,2) 288 genes (Sample 3)	Xenium	https://www.10xgenomics.com/products/xenium-in-situ/preview-dataset-human-breast
HP	Human	Pancreas	single-cell	1	140,702 cells	377 genes	Xenium	https://www.10xgenomics.com/datasets/ffpe-human-pancreas-with-xenium-multimodal-cell-segmentation-1-standard
HN	Human	Heart	single-cell	1	26,366 cells	377 genes	Xenium	https://www.10xgenomics.com/datasets/human-heart-data-xenium-human-multi-tissue-and-cancer-panel-1-standard
DLPFC	Human	Cortex	55μm	12	3,460-4,789 spots	33,538 genes	Visium	http://spatial.libd.org/spatialLIBD
HER2ST	Human	breast cancer	100μm	32	613 spots (Section $H1$) 441 spots (Section $G1$)	15,029 genes (Section $H1$) 14,992 genes (Section $G1$)	ST	https://github.com/almaan/her2st
MBC	Mouse	Brain (coronal)	55μm	1	2,235 spots	19.465 genes	Visium	https://www.10xgenomics.com/datasets/mouse-brain-coronal-section-2-ffpe-2-standard
MBS	Mouse	Brain (sagittal)	55μm	1	3,289 spots	32,285 genes	Visium	https://www.10xgenomics.com/datasets/mouse-brain-serial-section-2-sagittal-posterior-1-standard
MBHD	Mouse	Brain	8μm	1	453,820 squares	17,797 genes	Visium HD	https://www.10xgenomics.com/datasets/visium-hd-cytassist-gene-expression-mouse-brain-fresh-frozen
HBCHD	Human	Breast cancer	8μm	1	472,859 squares	17,527 genes	Visium HD	https://www.10xgenomics.com/datasets/visium-hd-cytassist-gene-expression-human-breast-cancer-fresh-frozen

Table S2. Accuracy of spatial domains identified from predicted data using scstGCN and iStar across all sections in DLPFC datasets. The data predicted by scstGCN consistently outperforms that predicted by iStar in terms of the Adjusted Rand Index (ARI), across all evaluations using the three different spatial clustering methods: AVGN, STAGATE, and GraphST.

**AVGN**	151507	151508	151509	151510	151669	151670	151671	151672	151673	151674	151675	151676
scstGCN	0.47	0.48	0.45	0.37	0.32	0.17	0.35	0.68	0.54	0.54	0.46	0.43
iStar	0.36	0.31	0.33	0.31	0.24	0.17	0.18	0.46	0.40	0.41	0.30	0.29
**STAGATE**	151507	151508	151509	151510	151669	151670	151671	151672	151673	151674	151675	151676
scstGCN	0.28	0.25	0.35	0.32	0.26	0.30	0.37	0.53	0.33	0.46	0.27	0.31
iStar	0.25	0.22	0.29	0.30	0.22	0.25	0.22	0.40	0.28	0.41	0.23	0.33
**GraphST**	151507	151508	151509	151510	151669	151670	151671	151672	151673	151674	151675	151676
scstGCN	0.32	0.28	0.36	0.30	0.24	0.13	0.36	0.58	0.24	0.38	0.37	0.41
iStar	0.26	0.21	0.32	0.27	0.20	0.18	0.24	0.41	0.21	0.26	0.29	0.35

Table S3. TLS (Tertiary lymphoid structure) marker genes from [1].

TLS marker genes	Celltype labels
CD4	Endothelial
CD8A	T cells
CD74	Myeloid
CD79A	Plasmablasts
IL7R	T cells
ITGAE	N/A
CD1D	N/A
CD3D	T cells
CD3E	T cells
CD8B	T cells
CD19	B cells
CD22	B cells
CD52	T cells
CD79B	B cells
CR2	Myeloid
CXCL13	N/A
CXCR5	B cells
FCER2	B cells
MS4A1	B cells
PDCD1	T cells
PTGDS	CAFs
TRBC2	T cells

Table S4. Comprehensive evaluation of the performance of GCN modules for establishing complex communication relationships between neighboring cells. **"Increased proportion (1)"** represents the percentage of performance improvement of scstGCN compared to scstGCN-GCN (meaning that replace GCN module with a feed-forward neural network consisting of four linear layers with 512 nodes in each layer). **"Increased proportion (2)"** represents the percentage of performance improvement of iStar+GCN compared to iStar (meaning that introduce GCN module to downstream method of iStar).

**Mean prediction error**	HBC_S1R1		HBC_S1R2		HBC_S2		MBHD		HBCHD	
	RMSE	SSIM	RMSE	SSIM	RMSE	SSIM	RMSE	SSIM	RMSE	SSIM
scstGCN	0.0420	0.7579	0.0419	0.7835	0.0495	0.7395	0.0462	0.7919	0.0507	0.7692
scstGCN-GCN	0.0642	0.6564	0.0618	0.6934	0.0699	0.6412	0.0745	0.6182	0.1040	0.5381
iStar+GCN	0.0435	0.7238	0.0429	0.7609	0.0509	0.7296	0.0558	0.7745	0.0573	0.7422
iStar	0.0787	0.5715	0.0660	0.6853	0.0736	0.6379	0.0881	0.5735	0.1267	0.4874
Increased proportion (1)	34.5%	15.5%	32.2%	13.0%	29.2%	15.3%	38.0%	28.2%	51.9%	42.9%
Increased proportion (2)	44.7%	26.7%	35.0%	11.0%	30.6%	14.4%	37.0%	35.1%	54.9%	52.4%

**Median prediction error**	HBC_S1R1		HBC_S1R2		HBC_S2		MBHD		HBCHD	
	RMSE	SSIM	RMSE	SSIM	RMSE	SSIM	RMSE	SSIM	RMSE	SSIM
scstGCN	0.0389	0.7688	0.0389	0.8014	0.0481	0.7386	0.0447	0.7840	0.0489	0.7546
scstGCN-GCN	0.0602	0.6636	0.0605	0.7368	0.0665	0.6323	0.0612	0.6243	0.0914	0.5478
iStar+GCN	0.0391	0.7299	0.0406	0.7910	0.0484	0.7189	0.0480	0.7804	0.0545	0.7282
iStar	0.0736	0.5811	0.0632	0.7256	0.0679	0.6295	0.0679	0.6057	0.0986	0.5537
Increased proportion (1)	35.4%	15.9%	35.7%	8.8%	27.7%	16.8%	27.0%	25.6%	47.5%	37.8%
Increased proportion (2)	46.9%	25.6%	35.8%	9.0%	28.7%	14.2%	29.3%	28.9%	44.4%	31.7%

Table S5. The computational resource requirements comparison between scstGCN and other baselines for runtime and memory consumption of the MBHD data from Visium HD technology.

	scstGCN	iStar	XFuse	TESLA	STAGE
Time / Seconds	294	252	45,938	89,609	5,763
CPU memory Usage / MB	7,168	6,232	4,395	15,710	456
GPU memory Usage / MB	2,497	2,563	5,813	-	5,545

References

1. Daiwei Zhang, Amelia Schroeder, et al. Inferring super-resolution tissue architecture by integrating spatial transcriptomics with histology. *Nature Biotechnology*, pages 1–6, 2024.
2. Richard J Chen, Chengkuan Chen, et al. Scaling vision transformers to gigapixel images via hierarchical self-supervised learning. In *Proceedings of the IEEE/CVF Conference on Computer Vision and Pattern Recognition (CVPR)*, pages 16144–16155, 2022.
3. Ludvig Bergenstråhle, Bryan He, and Bothers. Super-resolved spatial transcriptomics by deep data fusion. *Nature Biotechnology*, 40(4):476–479, 2022.
4. Jian Hu, Kyle Coleman, et al. Deciphering tumor ecosystems at super resolution from spatial transcriptomics with tesla. *Cell Systems*, 14(5):404–417, 2023.
5. Shang Li, Kuo Gai, Kangning Dong, Yiyang Zhang, and Shihua Zhang. High-density generation of spatial transcriptomics with stage. *Nucleic Acids Research*, 52(9):4843–4856, 2024.
6. Edward Zhao, Matthew R Stone, et al. Spatial transcriptomics at subspot resolution with bayesspace. *Nature Biotechnology*, 39(11):1375–1384, 2021.
7. Minxing Pang, Kenong Su, and Mingyao Li. Leveraging information in spatial transcriptomics to predict super-resolution gene expression from histology images in tumors. *BioRxiv*, pages 2021–11, 2021.
8. Richard J Chen, Tong Ding, et al. Towards a general-purpose foundation model for computational pathology. *Nature Medicine*, 30(3):850–862, 2024.
